# Foliar and Soil Application of B, Zn, and Si Fertilizers Induce Defense Responses in Wheat Plants Against Bipolaris Leaf Blight (BpLB)

**DOI:** 10.1155/sci5/2974890

**Published:** 2025-09-29

**Authors:** Rezoana Karim Humaira, Md. Morshedul Islam, Shila Chakraborty, Md. Atiqur Rahman Khokon

**Affiliations:** ^1^Department of Plant Pathology, Bangladesh Agricultural University, Mymensingh, Bangladesh; ^2^Department of Agriculture, Teesta University, Rangpur, Bangladesh

**Keywords:** *Bipolaris sorokiniana*, management, micronutrients, resistance, *Triticum aestivum* L.

## Abstract

Bipolaris leaf blight of wheat is a serious biological obstacle that can be seen at any growth stage. Fertilizer management and micronutrient application have crucial roles in plant disease management. In this study, boron, zinc, and silicon fertilizer were applied in soil and sprayed on the foliage of wheat plants at the seedling, tillering, and booting stage to investigate their effect on the incidence and severity of leaf blight disease caused by *Bipolaris sorokiniana* and also evaluated the defense responses against the pathogen. Nine treatment combinations were applied in a susceptible wheat cultivar Kanchan. Vegetative parameters like the number of plants/pots, number of leaves/pots, and plant height (cm) are significantly promoted by micronutrient application at all growth stages. All combinations of micronutrient treatments expressively influenced disease parameters at 45 and 60 days after sowing (DAS) and yield contributing characters at 100 DAS. Yield components were assessed at 100 DAS. The lowest incidence and severity of leaf blight were found in *T*_7_, where 50% of the total micronutrients were applied to the soil and 0.30 g Zn and 0.30 g Si were applied on the foliage of the wheat plants. Total phenol content (401.79 μg/g), MDA (68.90 nmol/g FW), and H_2_O_2_ (82.36 nmol/g FW) were gradually increased after micronutrient applied and recorded highest in *T*_7_ at 21 days after treatment (DAT). Antioxidant enzymes like catalase (CAT) (2.97 mM min^−1^ g^−1^ FW) and ascorbate peroxidase (APX) (15.36 mM min^−1^ g^−1^ FW) were also found highest in *T*_7_. It is revealed that B, Zn, and Si can increase tolerance related to certain biochemical attributes, vegetative growth, and yield contributing characters of wheat.

## 1. Introduction

Wheat (*Triticum aestivum* L.), which belongs to the family Poaceae, is one of the most important staple foods for the majority of the world's total population [1, 2]. After rice, it is the second most significant cereal crop in Bangladesh and it is cultivated in 0.35 million ha areas with the production of 1.2 million tons [3]. The production of wheat is compromised by several factors, but disease is the most significant one. Some major wheat diseases are rust, smut, powdery mildew, bipolaris leaf blight (BpLB), common root rot, fusarium head blight, blast, and several viral, nematode, and bacterial diseases. These diseases desperately impact the yield and cause death of the plants [4]. Among these various diseases, leaf blight caused by *Bipolaris sorokiniana* is the most destructive one [5]. The pathogen is widely dispersed throughout the world's wheat-growing regions, and it is much more active in hot, humid climates like Bangladesh [6].

The economic impact of the disease is therefore strongest in countries, such as India, Bangladesh, Nepal, and Brazil [7]. In South Asia, yield loss due to *B. sorokiniana* has been reported to be 20%–30% in farmers' fields and experiment stations [4, 8]. Gupta et al. [6] estimated a 15%–25% loss in wheat yield due to leaf blight disease. In case of a severe attack, it may result in 100% yield loss [9]. Farmers generally apply different chemical pesticides to control the leaf blight disease of wheat. Indiscriminate application of chemical fungicides causes the development of resistant races of pathogens, destroying beneficial microorganisms and polluting the freshwater [4].

Micronutrients assist plants in overcoming pathogen attacks under stress conditions and also provide benefits to plants and adverse effects to pathogens [10]. Soil is crucial for the retention of micronutrients like copper (Cu), boron (B), chlorine (Cl), zinc (Zn), iron (Fe), manganese (Mn), silicon (Si), and molybdenum (Mo), which can mitigate plant disease severity by enhancing disease tolerance and pathogen resistance. These nutrients can sufficiently reduce diseases [11]. Micronutrients are directly involved in plant protection by activating metabolic regulators, and several enzymes such as catalase (CAT), ascorbate peroxidase (APX), and superoxide dismutase in their defense systems [12, 13]. Thus, all these directly and indirectly help in plant growth and development, plant metabolism [14], and the reduction of diseases and other disorders. Micronutrients frequently participate in redox reactions and act as cofactors in enzyme systems, in addition to having numerous other vital roles in plants. So, small quantities (< 100 ppm) of micronutrients can induce systemic acquired resistance against plant diseases [15, 16]. Boron (B)-mediated metabolic and physiological processes diminish the disease vulnerability in the plant system by strengthening cell wall structure, controlling cell membrane permeability, and phenolics or lignin metabolism [17]. Silicon (Si) accumulates mainly in epidermal cells, creating a physical barrier for fungal hyphae penetration into plant roots [18]. Zinc (Zn) diminished disease severity, which could be due to its direct toxic action on the pathogen [19].

Farmers in Bangladesh apply boron and zinc as fertilizer supplements to increase crop productivity, but there is no report on the utilization of micronutrients for management and developing resistance against BpLB of wheat in Bangladesh. However, the use of micronutrients against plant pathogens might be an eco-friendly approach for disease management and improving plant vegetative and yield parameters. Therefore, the present study aimed to assess the efficacy of soil and foliar applications of B, Zn, and Si, both alone and in combination, for the management of BpLB disease in wheat and to elucidate their role in inducing biochemical defense mechanisms.

## 2. Materials and Methods

### 2.1. Experimental Site

The experiments were conducted in the laboratory of bio-signaling, bio-active compounds, and bio-formulation and the net house of Professor Golam Ali Fakir Seed Pathology Centre, Department of Plant Pathology, Bangladesh Agricultural University, Mymensingh. Both laboratory and net house experiments were laid out in a completely randomized design (CRD) with nine treatments, maintaining three replications.

### 2.2. Seed Collection, Pot Soil Preparation, Seed Sowing, and Fertilization Procedure

Wheat seeds (cv. Kanchan) were collected from the Bangladesh Agricultural Research Institute (BARI), Gazipur. Healthy-looking wheat seeds were surface sterilized with sodium hypochlorite (1.5% w/v), washed several times with sterile water, and dried [20]. A total of 27 plastic pots (30 cm × 30 cm) were filled with 15 kg of field soil previously treated with 5% formalin solution [21]. The pot was the experimental unit (*n* = 3 per treatment). Recommended doses of urea, TSP, MoP, and gypsum were mixed with soil and kept for 2 days for mixing. Urea was added in three splits (a) 1/3 during soil preparation; (b) 1/3 after 4 weeks of sowing, i.e., at the tillering stage; and (c) the remaining 1/3 after 6 weeks of sowing, i.e., at the tillering stage. Then, dried sterilized seeds were sown in pot soil and care was taken as necessary [22]. Finally, after germination, 10 plants per pot were kept for the experiments, and the data presented in tables are the mean of the three pots.

### 2.3. Isolation, Preservation, and Inoculation of *Bipolaris sorokiniana* Isolates

Single conidium of the pathogen from the blotter plate was carefully taken off with the help of a needle and then transferred to the center of potato dextrose agar (PDA) medium and incubated at 24 ± 2°C for 7–10 days [23]. Then, pure culture was made. For inoculation, conidial suspension (10^4^ conidia/mL of water) was prepared following the CIMMYT method [24], and in each pot, 20 mL of the conidial suspension was sprayed with a self-compressed hand sprayer at the three-leaf stage [9]. The pots were covered with polythene bags for 36 h to get better inoculation.

### 2.4. Preparation and Application Procedure of Micronutrients in Soil and Foliage

During soil preparation, zinc sulfate (36% Zn), Solubor boron (20% B), and silicon dioxide (46.75% Si) were applied directly to 24 pots as per recommended treatments, and the other 3 pots were maintained for a control treatment. For foliar application, the treatment recommended dose of each micronutrient was mixed with 1000 mL of water and then sprayed on the foliar parts of the plants. The first foliar spray was done at the seedling stage (30 DAS), the second at the tillering stage (45 DAS), and the third at the booting stage (60 DAS). Here,

• *T*_0_ = Control
• *T*_1_ = Soil application of 100% of total micronutrients (B, Zn, Si @ 2 kg/ha, 4 kg/ha, 2 kg/ha).
• *T*_2_ = Soil application of 50% of total micronutrients (B, Zn, Si) + foliar application of 0.15 g B/pot.
• *T*_3_ = Soil application of 50% of total micronutrients (B, Zn, Si) + foliar application of 0.30 g Si/pot.
• *T*_4_ = Soil application of 50% of total micronutrients (B, Zn, Si) + foliar application of 0.30 g Zn/pot.
• *T*_5_ = Soil application of 50% of total micronutrients (B, Zn, Si) + foliar application of (0.15 g B + 0.30 g Si)/pot.
• *T*_6_ = Soil application of 50% of total micronutrients (B, Zn, Si) + foliar application of (0.15 g B + 0.30 g Zn)/pot.
• *T*_7_ = Soil application of 50% of total micronutrients (B, Zn, Si) + foliar application of (0.30 g Si + 0.30 g Zn)/pot.
• *T*_8_ = Soil application of 50% of total micronutrients (B, Zn, Si) + foliar application of (0.30 g Si + 0.30 g Zn + 0.15 g B)/pot.

### 2.5. Assessment of Disease Incidence and Disease Severity Caused by *B. sorokiniana*

Disease incidence was determined by using the formula of Kumari et al. [25]:

(1) $$\begin{array}{c} \text{Disease incidence}=\frac{\text{No}.\text{ of infected plants}}{\text{Total no}.\text{ of plant assessed}}\times 100. \end{array}$$

Disease severity was estimated on infected leaves after 7 days of inoculation using a 0–5 severity scale according to Shahbaz et al. [26] by following the formula of Kumari et al. [25]:

(2) $$\begin{array}{c} \text{Disease severity}=\frac{\text{Sum of all disease rating}}{\text{Sum of numerical ratings}\times \text{maximum disease grade}}\times 100. \end{array}$$

Ratings for BpLB severity on wheat [26].

### 2.6. Biochemical Changes Associated With Induced Resistance

#### 2.6.1. Determination of Reactive Oxygen Species (ROS)

To measure the total ROS content of leaves, two parameters, *viz.*, malondialdehyde (MDA) activity (nmol/g FW) and hydrogen peroxide (H_2_O_2_) activity (nmol/g FW), were assayed at 7, 14, and 21 DAT.

##### 2.6.1.1. Determination of MDA Activity

MAD activity was determined by the method of Heath and Packer [27]. The amount of MDA was calculated by using the following formula:

(3) $$\begin{array}{c} \text{MDA }\left({\text{nmol }g}^{-1}\text{ FW}\right)=1000\left[\frac{\left(\text{Abs }523-\text{Abs }600\text{ nm}\right)}{155}\right], \end{array}$$

where extinction coefficient of MDA = 155 mM^−1 ^cm^−1^.

##### 2.6.1.2. Determination of Hydrogen Peroxide (H_2_O_2_) Activity

Hydrogen peroxide (H_2_O_2_) was quantified according to Alexieva et al. [28] protocol. The amount of H_2_O_2_ was determined by the following formula:

(4) $$\begin{array}{c} H_{2}O_{2}\;\left(µ{\text{mole }g}^{-1}\text{ FW}\right)=\frac{\text{Absorbance}}{\text{Extinction Co}‐\text{efficient}}\times \text{Dilution factor}, \end{array}$$

where extinction coefficient of H_2_O_2_ = 0.28 μM^−1 ^cm^−1^.

#### 2.6.2. Determination of Total Phenol Content (TPC)

To measure the TPC in leaves following the application of treatments and to observe the changes over time, leaves were collected 7, 14, and 21 days following 30, 45, and 60 days after treatment. TPC was assessed according to a standard protocol developed by Singleton et al. [29] and Ding et al. [30].

#### 2.6.3. Determining Antioxidant Enzyme Activity

To quantify the antioxidant enzyme activity of leaves, two parameters, *viz.*, CAT activity (mM min^−1^ g^−1^ FW) and APX activity (μmol min^−1^ g^−1^ FW), were examined at 21 DAT.

##### 2.6.3.1. Determination of CAT Activity

CAT activity was determined by following the method of Aebi [31]. The activity of CAT was calculated from the decrease in absorbance per minute by using this formula:

(5) $$\begin{array}{c} \text{CAT }\left({\text{mM }\min}^{-1}{\text{⁣}g}^{-1}\text{ FW}\right)=\frac{\left(\text{Absorbance difference}/\min\right)\times \text{Dilution factor}\times 1000\;}{40\times 1000}, \end{array}$$

where extinction coefficient = 40 M^−1 ^cm^−1^ of CAT.

##### 2.6.3.2. Determination of APX Activity

APX activity was determined by following the method of Nakano and Asada [32]. The activity of APX was calculated from the decrease in absorbance per minute by using the following formula:

(6) $$\begin{array}{c} \text{APX }\left(µ{\text{mol }\min}^{-1}{\text{⁣}g}^{-1}\text{ FW}\right)=\frac{\left(\text{Absorbance difference}/\min\right)\times \text{Dilution factor}\times 1000\;}{2.8\times 1000}, \end{array}$$

where extinction coefficient = 2.8 M^−1 ^cm^−1^ of APX.

### 2.7. Data Recording and Analysis

The collected data on different parameters were analyzed statistically by using Minitab 18 software. The significance of the difference among the mean was calculated by Duncan's multiple range test (DMRT) when one-way ANOVA revealed significant differences. To find out the relation between vegetative and disease parameters with disease incidence (%) and severity (%), the correlation coefficient was analyzed using Minitab 18 software.

## 3. Results

### 3.1. Effect of Micronutrient Application on Vegetative Growth of Wheat Plants

Number of plants/pots: The number of plants/pots significantly varied in different treatments at 30 DAS ranging from 35.00 to 44.67 (Table 1). The highest number of plants (44.67) was recorded in *T*_7_ (soil application of 50% of total micronutrients [B, Zn, Si] + foliar application of [0.30 g Si + 0.30 g Zn]/pot), whereas the lowest number of plants (35.00) was found in *T*_0_ (control).

Number of leaves/pots: The number of leaves/pots was significantly diverse in different treatments of micronutrients at both 30 and 45 DAS (Table 1). The highest number of leaves (155.67) was found in *T*_7_ (soil application of 50% of total micronutrients [B, Zn, Si] + foliar application of [0.30 g Si + 0.30 g Zn]/pot) at 45 DAS.

Plant height (cm): A gradual increment in plant height was found up to 60 DAS. Micronutrient application significantly influences the plant height at every growth stage (Table 1). The highest plant height was recorded in *T*_4_ (soil application of 50% of total micronutrients [B, Zn, Si] + foliar application of 0.30 g Zn/pot) followed by *T*_3_ (soil application of 50% of total micronutrients [B, Zn, Si] + foliar application of 0.30 g Si/pot), *T*_7_ (Soil application of 50% of total micronutrients [B, Zn, Si] + foliar application of [0.30 g Si + 0.30 g Zn]/pot), and *T*_8_ (soil application of 50% of total micronutrients [B, Zn, Si] + foliar application of [0.30 g Si + 0.30 g Zn + 0.15 g B]/pot).

### 3.2. Effect of Micronutrient Application on Different Disease Parameters Developed on Wheat Plants

Different disease parameters, *viz*., no. of infected tiller/pot, no. of infected plants/pot, no. of infected leaves/pot, and % leaf area infection, were assessed at 45 and 60 DAS (Table 2). No symptoms were noticed in the inoculated plants at 30 DAS. All disease parameters showed significant variation in different micronutrient treatments at both 45 and 60 DAS. At 60 DAS, all disease parameters were higher compared to 45 DAS.

Number of infected tillers/pots: The number of infected tillers/pots was higher (12.00) at *T*_0_ (control) which was identical (12.00) to *T*_1_ (B, Zn, Si @ 2 kg/ha, 4 kg/ha, and 2 kg/ha) and the lowest (9.00) in *T*_7_ (soil application of 50% of total micronutrients [B, Zn, Si] + foliar application of [0.30 g Si + 0.30 g Zn]/pot) at 45 DAS. A similar trend was found at 60 DAS, and control showed the highest no. of infected tiller/pot (16.00) and lowest in *T*_7_ (10.00).

Number of infected plants/pots: Significant differences were observed among the different treatments on the number of infected plants/pots of wheat at 45 and 60 DAS (Table 2). Slightly higher number of infected plants/pot were found at 60 DAS compared to 45 DAS. At 45 DAS, the highest and statistically similar no. of infected plants/pot (35.67) was recorded in *T*_1_ (soil application of 100% of total micronutrients [B, Zn, Si] @ [2 kg/ha, 4 kg/ha and 2 kg/ha]) and *T*_3_ (soil application of 50% of total micronutrients [B, Zn, Si] + foliar application of 0.30 g Si/pot), whereas the lowest no. of infected plants/pot (26.33) was recorded in *T*_7_ (soil application of 50% of total micronutrients [B, Zn, Si] + foliar application of [0.30 g Si + 0.30 g Zn]/pot). At 60 DAS, the highest no. of infected plants/pot (39.67) was recorded in *T*_3_ (soil application of 50% of total micronutrients [B, Zn, Si] + foliar application of 0.30 g Si/pot), whereas the lowest number of infected plants/pot (30.33) was found *T*_7_ (soil application of 50% of total micronutrients [B, Zn, Si] + foliar application of [0.30 g Si + 0.30 g Zn]/pot).

Number of infected leaves/pots: No. of infected leaves/pots are higher at 60 DAS than 45 DAS (Table 2). *T*_7_ (soil application of 50% of total micronutrients [B, Zn, Si] + foliar application of [0.30 g Si + 0.30 g Zn]/pot) yielded the lowest no. of infected leaves/pot at both 45 (76.67) and 60 (80.67) DAS. The control showed the highest no. of infected leaves/pots at both stages.

Percent leaf area infection: Different treatments of micronutrient application showed a significant influence on the percent leaf area infected at 45 and 60 DAS (Table 2). A slightly increased percent leaf area infected was found at 60 DAS. At both growth stages, control showed the highest percent leaf area infection. At 60 DAS, statistically similar and higher percent leaf area infected were found in *T*_0_ (27.00%), *T*_2_ (26.67%), *T*_4_ (27.00%), and *T*_6_ (26.33%). At both growth stages, *T*_7_ (soil application of 50% of total micronutrients [B, Zn, Si] + foliar application of [0.30 g Si + 0.30 g Zn]/pot) showed the lowest percent leaf area infected.

### 3.3. Effect of Micronutrient Application on Yield Contributing Parameters of Wheat

Number of total spikelets/pots: Different treatments of micronutrients showed significant variation in no. of total spikelets/pots at 100 DAS ranging from 27.00 to 40.67 (Table 3). The lowest no. of total spikelets/pot was recorded in the control plot, and the highest was in *T*_7_ (Soil application of 50% of total micronutrients [B, Zn, Si] + foliar application of [0.30 g Si + 0.30 g Zn]/pot).

Length of spikelet (cm): At 100 DAS, length of the spikelet varied significantly in different treatments of micronutrients (Table 3). The highest length of the spikelet (22.86 cm) was recorded in *T*_7_ (soil application of 50% of total micronutrients [B, Zn, Si] + foliar application of [0.30 g Si + 0.30 g Zn]/pot) and the lowest length of the spikelet (12.70 cm) was recorded in *T*_0_ (control).

Number of healthy spikelets/pots: At 100 DAS, healthy spikelets/pots ranged from 16.00 to 30.00 (Table 3). Micronutrient application significantly influenced the no. of healthy spikelets/pot. The highest healthy spikelets/pot (30.00) was recorded in *T*_7_ (soil application of 50% of total micronutrients [B, Zn, Si] + foliar application of [0.30 g Si + 0.30 g Zn]/pot). The lowest healthy spikelets/pot (16.00) were recorded in *T*_0_ (control).

Healthy-looking grains/pots: Micronutrient application significantly influenced the healthy-looking grains/pots after harvest (Table 3). The highest no. of healthy-looking grains/pot (695.33) was recorded in *T*_7_ (soil application of 50% of total micronutrients [B, Zn, Si] + foliar application of [0.30 g Si + 0.30 g Zn]/pot). The lowest no. of healthy grains/pot (567.33) was recorded in *T*_0_ (control).

Number of infected grains/pots: Significant variation was found in no. of infected grains/pots after harvest (Table 3). The highest infected grains/pots (24.67) were recorded in *T*_0_ (control). The lowest infected grains/pots (13.33) were recorded in *T*_7_ (soil application of 50% of total micronutrients [B, Zn, Si] + foliar application of [0.30 g Si + 0.30 g Zn]/pot) which was statistically similar to *T*_2_ (14.67), *T*_6_ (14.33), and *T*_8_ (14.67).

Total grains/pots: The total no. of grains/pots significantly varied in different micronutrient treatments after harvest (Table 3). The highest no. of total grains/pot (708.67) was recorded in *T*_7_ (soil application of 50% of total micronutrients [B, Zn, Si] + foliar application of [0.30 g Si + 0.30 g Zn]/pot) and the lowest no. of total grain/pot (592.00) was recorded in *T*_0_ (control).

### 3.4. Effect of Micronutrient Application on the Incidence and Severity of BpLB of Wheat at Different Growth Stages

**Figure 1:**
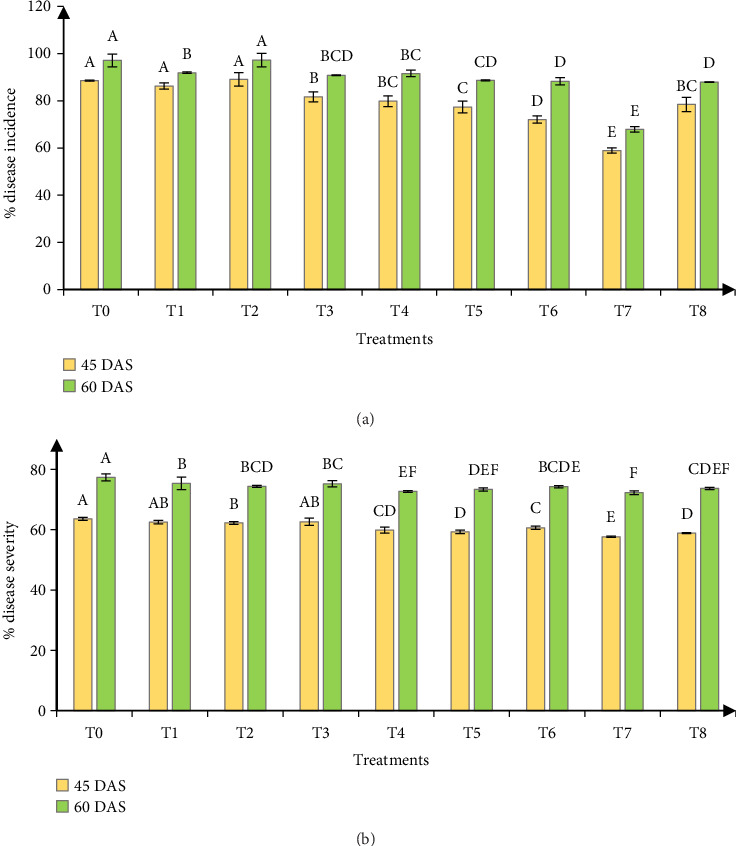
Effect of micronutrient application on the (a) incidence and (b) severity of bipolaris leaf blight (*Bipolaris sorokiniana*) of wheat at tillering (45 DAS) and booting (60 DAS) growth stages. Here, *T*_0_ = control, *T*_1_ = soil application of 100% of total micronutrients (B, Zn, Si) @ (2 kg/ha, 4 kg/ha, and 2 kg/ha), *T*_2_ = soil application of 50% of total micronutrients (B, Zn, Si) + foliar application of 0.15 g B/pot, *T*_3_ = soil application of 50% of total micronutrients (B, Zn, Si) + foliar application of 0.30 g Si/pot, *T*_4_ = soil application of 50% of total micronutrients (B, Zn, Si) + foliar application of 0.30 g Zn/pot, *T*_5_ = soil application of 50% of total micronutrients (B, Zn, Si) + foliar application of (0.15 g B + 0.30 g Si)/pot, *T*_6_ = soil application of 50% of total micronutrients (B, Zn, Si) + foliar application of (0.15 g B + 0.30 g Zn)/pot, *T*_7_ = soil application of 50% of total micronutrients (B, Zn, Si) + foliar application of (0.30 g Si + 0.30 g Zn)/pot, *T*_8_ = soil application of 50% of total micronutrients (B, Zn, Si) + foliar application of (0.30 g Si + 0.30 g Zn + 0.15 g B)/pot.

Application of micronutrients significantly influenced the disease incidence and severity of BpLB at the tillering (45 DAS) and booting (60 DAS) stage (Figure 1). With the progress of time, the incidence and severity of the disease increased. At 60 DAS, the highest disease incidence (97.29%) was recorded in *T*_0_ (control) followed by (97.14%) *T*_2_ (soil application of 50% of total micronutrients [B, Zn, Si] + foliar application of 0.15 g B/pot) and the treatment *T*_7_ (soil application of 50% of total micronutrients [B, Zn, Si] + foliar application of [0.30 g Si + 0.30 g Zn]/pot) showed the lowest disease incidence (67.91%). At 60 DAS, the highest disease severity (77.34%) was recorded in *T*_0_ (control). On the other hand, the lowest disease severity (72.25%) was recorded in *T*_7_ (soil application of 50% of total micronutrients [B, Zn, Si] + foliar application of [0.30 g Si + 0.30 g Zn]/pot).

### 3.5. Induction of Resistance Against BpLB of Wheat by Soil and Foliar Application of Micronutrients

#### 3.5.1. Effect of Application of Micronutrients on ROS Production in Wheat Leaves

Application of micronutrients led to a significant increase in ROS content over time in this experiment. To measure the total ROS content of leaves, two parameters, *viz.*, MDA activity (nmol/g FW) and hydrogen peroxide (H_2_O_2_) (μmol/g FW) were observed (Table 4). The highest amount of MDA (68.90 nmol/g FW) was recorded in *T*_7_ (soil application of 50% of total micronutrients [B, Zn, Si] + foliar application of [0.30 g Si + 0.30 g Zn]/pot) at 21 DAT, and the lowest (33.82 nmol/g FW) was found in the control treatment. On the other hand, the H_2_O_2_ content of the wheat plants was recorded as highest for treatment *T*_7_ (82.36 μmol/g FW) at 21 DAT. So, it is very clear that in most of the cases, both the MDA and H_2_O_2_ contents were highest in *T*_7_ treatment.

#### 3.5.2. Effect of Application of Micronutrients on TCP and Antioxidant Enzyme Activity in Wheat Leaves

TPC (μg/g) in wheat leaves accumulated by application of micronutrients significantly increased at 7, 14, and 21 DAT (Table 5). Among the all treatment combination, the highest phenol contents of 305.38 μg/g at 7 DAT, 357.95 μg/g at 14 DAT, and 401.79 μg/g at 21 DAT were recorded in *T*_7_ (soil application of 50% of total micronutrients [B, Zn, Si] + foliar application of [0.30 g Si + 0.30 g Zn]/pot) and the lowest phenol contents were found in control. So, the TPC gradually increased with time.

Two different enzymes, *viz.*, CAT and APX activity, were observed in different micronutrient-treated wheat leaves (Table 5). The CAT activity profile ranged from 0.46 to 2.97 mM min^−1^ g^−1^. The CAT activity was significantly higher in *T*_7_ (soil application of 50% of total micronutrients [B, Zn, Si] + foliar application of [0.30 g Si + 0.30 g Zn]/pot) (2.97 mM min^−1^ g^−1^). The lowest induction level was recorded in *T*_0_ (0.46 mM min^−1^ g^−1^), where no micronutrient was applied.

APX activity profile ranged from 5.00 to 15.36 μmol min^−1^ g^−1^ FW. Significantly highest APX activity (15.36 μmol min^−1^ g^−1^ FW) was recorded in *T*_7_ (soil application of 50% of total micronutrients [B, Zn, Si] + foliar application of [0.30 g Si + 0.30 g Zn]/pot) followed by (14.64 μmol min^−1^ g^−1^ FW) in *T*_5_ (soil application of 50% of total micronutrients [B, Zn, Si] + foliar application of [0.15 g B + 0.30 g Si]/pot). On the contrary, the lowest amount of APX (5.00 μmol min^−1^ g^−1^ FW) was found in *T*_0_ (control).

### 3.6. Correlation Matrix Analyses

**Figure 2:**
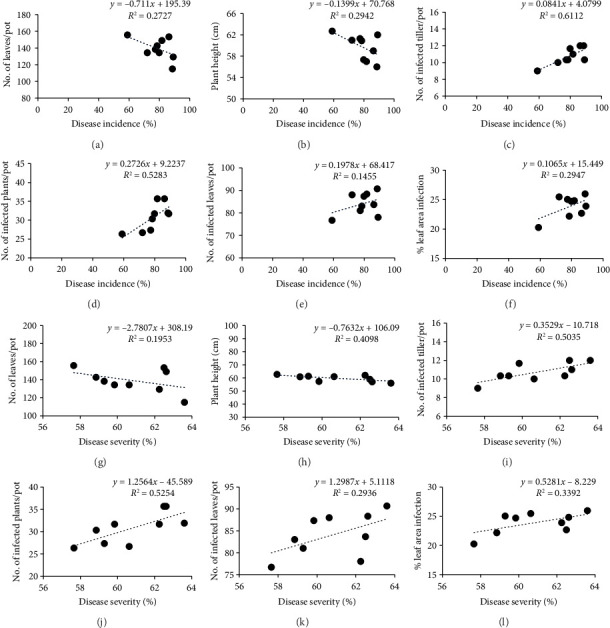
Correlation matrix representing the relationships among vegetative growth parameters, disease parameters, percent disease incidence (a–f), and percent disease severity (g–l) evaluated at 45 days after sowing (DAS).

**Figure 3:**
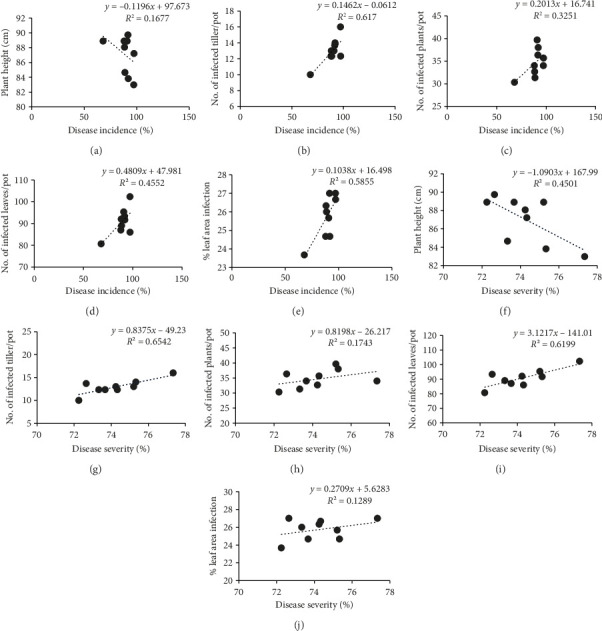
Correlation matrix representing the relationships among vegetative growth parameters, disease parameters, percent disease incidence (a–e), and percent disease severity (f–j) evaluated at 60 days after sowing (DAS).

A correlation matrix was developed to analyze whether vegetative parameters and disease parameters have any relation with disease incidence and severity (Figures 2 and 3). The analysis revealed that a significant negative correlation exists among vegetative parameters and a positive correlation exists among disease parameters with the incidence and severity of BpLB disease at 45 and 60 DAS. This finding confirms that the application of micronutrients has an impact on the physiological processes of wheat plants at various growth stages. Consequently, it reduces the occurrence and severity of leaf blight disease by enhancing the plant's tolerance to the disease, ultimately leading to improved yield of wheat (Table 6).

## 4. Discussion

Micronutrients are required by plants in trace amounts, but they are very important for plant growth, development, and protecting themselves against invading pathogens. Generally, micronutrients are applied during land preparation, but in the present experiment, micronutrients were applied both in land preparation and sprayed on the foliage to control the BpLB by improving the innate immunity system of wheat plants. It is evidenced from the present experiment that micronutrient application at the tillering stage (30 DAS) can significantly increase the no. of plants/pot by *T*_7_ (44.67) compared to the control (35). Significantly higher no. of plants were also found by micronutrient application reported by Nadim et al. [33], which supports the present findings. The vegetative parameters were significantly increased by all treatments of micronutrients. Still, the highest effect was recorded in treatment *T*_7_, where half of the total micronutrients were applied in the soil and the rest of the zinc and silicon were applied to the foliage of the wheat plant. The findings of the present study indicate that split foliar application of micronutrients enhances vegetative growth. A similar kind of research was also conducted by Singh et al. [34] on the effect of different micronutrients on plant growth, yield, and flower bud quality of broccoli (*Brassica oleracea* var *italica*). Disease parameters were significantly reduced by micronutrient application at both growth stages, suggesting that leaf blight can be effectively managed by split application of micronutrients in soil and foliage. According to Simoglou and Dordas [35], foliar application of boron to wheat plants resulted in a significant reduction in several lesions of tan spots of wheat. Boron significantly affected the number of lesions per leaf during booting and milk stages. Yield contributing parameters showed significant differences in different micronutrient treatments. Although all treatments of micronutrients showed significant differences, the highest effect was recorded in the *T*_7_ treatment. The findings of the present study indicate that foliar application of micronutrients reduces disease parameters. A similar kind of research was also conducted by Singh et al. [34] on the effect of different micronutrients on plant growth, yield, and flower bud quality of broccoli (*Brassica oleracea* var *italica*), and they observed the effect of micronutrients on postharvest observation and quality parameters of broccoli and found that these parameters were significantly higher at the micronutrient-treated plants compared to untreated plant which is similar to present experiment.

Incidence and severity of BpLB significantly differed in different treatments of micronutrients at 45 and 60 DAS. The disease was first noticed at 45 DAS and continued up to 60 DAS, indicating that the disease was less damaging at the early growth stage (30 DAS). Disease incidence and severity levels decreased in the micronutrient-treated plant compared to the untreated plant and providing evidence that micronutrients can bring some changes in the physiology of wheat plants to lower the incidence and severity of leaf blight. Elmer and Datnoff [36] reported that silicon (Si) application is a viable method of enhancing the resistance of several plant species to root and foliar pathogens and suppressing plant diseases effectively as a fungicide. Singh et al. [37] also reported that Zn has an adverse effect on the growth and spore germination of *B. sorokiniana*, and it significantly reduces the disease caused by the pathogen. It is reported by some scientists that boron (B) provides direct strength and stability for the cell wall and has a beneficial effect on reducing disease severity, which contributes to plant resistance and tolerance [38–40].

To understand the mechanism of micronutrients for reducing the incidence and severity of BpLB of wheat, three important indicators related to the defense mechanism against disease, *viz.*, production of ROS, TPC, and activity of antioxidant enzymes, were quantified spectrophotometrically and found significantly increased compared to control. Production of ROS such as superoxide anion, hydrogen peroxide, and hydroxyl radical is the first line of defense of plants against pathogens [41]. ROS have direct antimicrobial activities which reduce pathogen viability. MDA and hydrogen peroxide (H_2_O_2_) production were quantified to get the cumulative ROS production after the application of micronutrients.

MDA and H_2_O_2_ content gradually increased with time and remained significantly high in *T*_7_ up to 21 DAT. Moreover, the ROS content in *T*_1_ (100% micronutrients were applied in soil) was comparatively lower than *T*_7_, indicating that soil and foliar application of B, Si, and Zn can influence plant physiology to accumulate more ROS than only soil application. Plants use ROS as signaling molecules to initiate defensive mechanisms against infections. Through lignification, they reinforce cell walls, trigger a hypersensitive reaction that results in localized cell death, and induce genes linked to defense. Furthermore, ROS reduces disease incidence by limiting pathogen transmission, enhancing the generation of antimicrobial compounds, and interacting with hormones [42, 43]. Torres et al. [44] reported that the production of ROS was directly involved in the mechanisms that stopped pathogen growth which complies with the findings of the present research work. It is also similar to the report by Kotchoni and Gachomo [45], who reported that the cellular ROS level triggers beneficial effects on plant cells responding to pathogen attack.

The constitutional level of total phenol did not show a substantial increase within a week, but gradually increased, and the highest phenol content was recorded in the *T*_7_ treatment. Likewise, ROS production and TPC gradually increased over time. It can be assumed that as long as the ROS concentration is high, the total phenol concentration is also high. The present experimental finding reveals that soil application and foliar spray of micronutrients increase phenol content. Gupta et al. [46] revealed that soluble Si promotes the quick deposition of phenolics at infection sites, serving as a general defense against pathogen invasion. Taoutaou et al. [47] identified some phenolic compounds determining potato resistance against late blight. The research findings of Yao et al. [48] revealed that phenolic compounds are important plant secondary metabolites for plant defense. The application of micronutrients increased phenolic compounds by 40% in rice grains compared to the control [49]. These findings corroborate the findings of the present research work.

The necessity of micronutrients not only promotes the growth and development of plants but also acts as cofactors for the activation of several antioxidant enzymes. The activity of antioxidant enzymes may alter their mechanism of action under the micronutrient-deficient condition which in turn acts as sensitivity to environmental stress [50]. Shetty et al. [51] and Polanco et al. [52] found that Si application also increases the activity of antioxidative enzymes involved in plant defense such as peroxidase, polyphenol oxidase, phenylalanine ammonia lyase, and lipoxygenase, which are considered as chemical barriers to pathogen entry in the host plant. Fleck et al. [53] found that enhanced activities of antioxidative enzymes in rice leaves and increased systemic acquired resistance due to Si addition can reduce the severity of rice sheath blight disease. In the present experiment, soil and foliar application of micronutrients increases H_2_O_2_ production by the increased activity of antioxidant enzymes like CAT and APX to keep the ROS at homeostasis conditions. However, with this oxidative burst, TPC significantly increased in leaves which may be responsible for reducing BpLB incidence and severity in wheat plants. Therefore, micronutrients like B and Si can be applied to the soil and foliage as a split form to manage the BpLB in wheat.

## 5. Limitations

We acknowledge that this was a controlled pot experiment and that field validation is necessary to confirm the efficacy and economic viability of the *T*_7_ (soil application of 50% of total micronutrients [B, Zn, Si] + foliar application of [0.30 g Si + 0.30 g Zn]/pot) treatment under varying environmental conditions and soil types.

## 6. Conclusion

This study highlights the role of micronutrient supplementation in mitigating BpLB of wheat through enhanced host resistance and improved yield performance. Among the treatments, Zn, B, and Si demonstrated positive effects on biochemical defense traits, but the combined split application of Zn and Si (*T*_7_) proved most effective, resulting in substantial reductions in disease severity and a notable increase in total grains per pot. These findings suggest that targeted micronutrient management can serve as an environmentally friendly and sustainable alternative to sole chemical control. The observed improvement in resistance-related biochemical attributes further supports the physiological basis of micronutrient-induced protection. However, the current results are based on pot experiments, and validation under field conditions across multiple cultivars and agroecological zones is essential to establish robust recommendations. It is also imperative to explore the feasibility of micronutrient application and bio-control agents simultaneously to have some synergistic effect in managing BpLB disease more effectively. Therefore, a split application of Zn and Si (*T*_7_) can be recommended after trial in different agroecological zones.

## Figures and Tables

**Table 1:** Effect of micronutrients on vegetative growth of wheat at 30, 45, and 60 DAS in the net house under artificial disease pressure by *B. sorokiniana*.

Treatments	30 DAS			45 DAS		60 DAS
	No. of plants/pots	No. of leaves/pots	Plant height (cm)	No. of leaves/pots	Plant height (cm)	Plant height (cm)
*T*_0_	35.00^g^	108.33^g^	36.67^e^	115.00^h^	56.00^f^	82.98^g^
*T*_1_	41.33^c^	143.33^b^	38.00^d^	153.33^b^	59.00^d^	83.82^f^
*T*_2_	36.67^f^	119.33^f^	39.00^c^	129.33^g^	62.00^ab^	87.21^d^
*T*_3_	43.67^b^	124.33^e^	36.67^e^	149.00^c^	57.00^e^	88.90^b^
*T*_4_	39.67^d^	106.67^h^	41.00^ab^	134.33^f^	57.33^e^	89.73^a^
*T*_5_	35.33^g^	128.00^d^	40.67^b^	138.33^e^	61.30^bc^	84.66^e^
*T*_6_	37.00^f^	124.67^e^	40.33^b^	134.33^f^	61.00^c^	88.06^c^
*T*_7_	44.67^a^	147.33^a^	41.67^a^	155.67^a^	62.70^a^	88.90^b^
*T*_8_	38.67^e^	133.33^c^	41.00^ab^	142.67^d^	60.89^c^	88.90^b^

Level of significance	∗∗	∗∗	∗∗	∗∗	∗∗	∗∗

CV (%)	1.30	0.50	1.37	0.43	0.79	0.32

*Note:* ∗∗ = 1% level of significance. (*T*_0_ = control, *T*_1_ = soil application of 100% of total micronutrients [B, Zn, Si] @ [2 kg/ha, 4 kg/ha and 2 kg/ha], *T*_2_ = soil application of 50% of total micronutrients [B, Zn, Si] + foliar application of 0.15 g B/pot, *T*_3_ = soil application of 50% of total micronutrients [B, Zn, Si] + foliar application of 0.30 g Si/pot, *T*_4_ = soil application of 50% of total micronutrients [B, Zn, Si] + foliar application of 0.30 g Zn/pot, *T*_5_ = soil application of 50% of total micronutrients [B, Zn, Si] + foliar application of [0.15 g B + 0.30 g Si]/pot, *T*_6_ = soil application of 50% of total micronutrients [B, Zn, Si] + foliar application of [0.15 g B + 0.30 g Zn]/pot, *T*_7_ = soil application of 50% of total micronutrients [B, Zn, Si] + foliar application of [0.30 g Si + 0.30 g Zn]/pot, *T*_8_ = soil application of 50% of total micronutrients [B, Zn, Si] + foliar application of [0.30 g Si + 0.30 g Zn + 0.15 g B]/pot). a, b, c, d, e, f, g, h indicate significant differences among treatments based on statistical analysis. Specifically, mean separation was performed using the Least Significant Difference (LSD) test at *p*<0.05 (Statistix 10). Values sharing the same letter are not significantly different, whereas values with different letters differ significantly at the 5% probability level.

Abbreviation: CV = coefficient of variation.

**Table 2:** Effect of micronutrient application on disease parameters developed on wheat plants at 45 and 60 DAS in the net house under artificial disease pressure by *B. sorokiniana*.

Treatments	45 DAS				60 DAS			
	No. of infected tillers/pots	No. of infected plants/pots	No. of infected leaves/pots	% leaf area infection	No. of infected tillers/pots	No. of infected plants/pots	No. of infected leaves/pots	% leaf area infection
*T*_0_	12.00^a^	31.90^b^	90.67^a^	25.96^a^	16.00^a^	34.00^d^	102.33^a^	27.00^a^
*T*_1_	12.00^a^	35.67^a^	83.67^c^	22.67^dc^	14.00^b^	38.00^b^	91.67^d^	24.67^bc^
*T*_2_	10.33^cd^	31.67^b^	78.00^e^	23.89^cd^	12.33^d^	35.67^c^	86.00^g^	26.67^a^
*T*_3_	11.00^bc^	35.67^a^	88.33^b^	24.82^abc^	13.00^c^	39.67^a^	95.33^b^	25.67^ab^
*T*_4_	11.67^ab^	31.67^b^	87.33^b^	24.69^bc^	13.67^b^	36.33^c^	93.33^c^	27.00^a^
*T*_5_	10.33^cd^	27.33^c^	81.00^d^	25.03^abc^	12.33^d^	31.33^f^	89.00^e^	26.00^ab^
*T*_6_	10.00^d^	26.67^c^	88.00^b^	25.45^ab^	13.00^c^	32.67^e^	92.00^d^	26.33^a^
*T*_7_	9.00^e^	26.33^c^	76.67^e^	20.25^f^	10.00^e^	30.33^f^	80.67^h^	23.67^c^
*T*_8_	10.33^cd^	30.33^b^	83.00^c^	22.17^e^	12.33^d^	34.00^d^	87.00^f^	24.67^bc^

Level of significance	∗∗	∗∗	∗∗	∗∗	∗∗	∗∗	∗∗	∗∗

CV (%)	4.74	2.07	0.94	2.99	2.96	1.15	0.59	3.42

*Note:* ∗∗ = 1% level of significance. a, b, c, d, e, f, g, h indicate significant differences among treatments based on statistical analysis. Specifically, mean separation was performed using the Least Significant Difference (LSD) test at *p*< 0.05 (Statistix 10). Values sharing the same letter are not significantly different, whereas values with different letters differ significantly at the 5% probability level.

Abbreviation: CV = coefficient of variation.

**Table 3:** Effect of micronutrients on yield parameters of wheat plants at 100 DAS in the net house under artificial disease pressure by *B. sorokiniana*.

Treatments	Total spikelets/pots	Length of spikelets (cm)	Healthy spikelets/pots	Healthy-looking grains/pots	Infected grains/pots	Total grains/pots
*T*_0_	27.00^f^	12.70^g^	16.00^f^	567.33^i^	24.67^a^	592.00^h^
*T*_1_	29.33^e^	15.24^f^	21.33^e^	685.67^b^	18.00^c^	703.67^b^
*T*_2_	29.67^e^	16.13^e^	22.67^d^	579.33^g^	14.67^d^	594.00^g^
*T*_3_	31.33^d^	17.76^d^	23.00^d^	629.33^e^	18.67^c^	648.00^e^
*T*_4_	29.67^e^	15.24^f^	21.00^e^	601.33^f^	19.33^bc^	620.66^f^
*T*_5_	33.67^c^	20.32^c^	23.67^c^	573.00^h^	20.67^b^	593.67^gh^
*T*_6_	35.00^b^	21.67^b^	27.00^b^	665.00^c^	14.33^d^	679.33^c^
*T*_7_	40.67^a^	22.86^a^	30.00^a^	695.33^a^	13.33^d^	708.67^a^
*T*_8_	32.00^d^	20.67^c^	23.00^d^	635.33^d^	14.67^d^	650.00^d^

Level of significance	∗∗	∗∗	∗∗	∗∗	∗∗	∗∗

CV (%)	1.80	1.78	1.44	0.158	5.69	0.15

*Note:* ∗∗ = 1% level of significance. a, b, c, d, e, f, g, h, i indicate significant differences among treatments based on statistical analysis. Specifically, mean separation was performed using the Least Significant Difference (LSD) test at *p*< 0.05 (Statistix 10). Values sharing the same letter are not significantly different, whereas values with different letters differ significantly at the 5% probability level.

Abbreviation: CV = coefficient of variation.

**Table 4:** Effect of micronutrients on MDA and H_2_O_2_ content of wheat leaves.

Treatments	MDA (nmol/g FW)			H_2_O_2_ (μmol/g FW)		
	7 DAT	14 DAT	21 DAT	7 DAT	14 DAT	21 DAT
*T*_0_	16.10^i^	29.30^i^	33.82^i^	51.54^g^	54.95^i^	70.29^g^
*T*_1_	20.93^h^	46.36^d^	62.46^c^	51.40^h^	71.99^d^	72.70^f^
*T*_2_	26.73^f^	43.14^e^	62.37^d^	51.54^g^	71.28^e^	74.83^e^
*T*_3_	44.44^a^	47.97^c^	61.18^e^	53.67^f^	66.03^f^	78.24^c^
*T*_4_	22.54^g^	40.25^h^	53.77^g^	60.91^d^	63.61^g^	77.53^d^
*T*_5_	33.16^d^	55.06^b^	64.07^b^	64.32^c^	75.82^b^	78.38^b^
*T*_6_	37.64^c^	42.82^f^	58.60^f^	68.01^b^	72.42^c^	78.38^b^
*T*_7_	38.96^b^	59.24^a^	68.90^a^	69.58^a^	79.37^a^	82.36^a^
*T*_8_	29.95^e^	41.86^g^	45.40^h^	58.78^e^	62.76^h^	69.86^h^

Level of significance	∗∗	∗∗	∗∗	∗∗	∗∗	∗∗

CV (%)	0.111	0.023	0.019	0.047	0.015	0.001

*Note:* ∗∗ = 1% level of significance. a, b, c, d, e, f, g, h, i indicate significant differences among treatments based on statistical analysis. Specifically, mean separation was performed using the Least Significant Difference (LSD) test at *p*< 0.05 (Statistix 10). Values sharing the same letter are not significantly different, whereas values with different letters differ significantly at the 5% probability level.

Abbreviation: CV = coefficient of variation.

**Table 5:** Effect of micronutrients on total phenol content (TCP) and antioxidant enzyme activity of wheat leaves.

Treatments	Total phenol (μg/g)			Enzyme content	
	7 DAT	14 DAT	21 DAT	Catalase (CAT) mM min^−1^ g^−1^	Ascorbate peroxidase (APX) μmol min^−1^ g^−1^ FW
*T*_0_	294.36^g^	297.69^h^	297.95^i^	0.46^i^	5.00^e^
*T*_1_	296.15^f^	300.00^e^	303.60^g^	1.95^f^	9.28^b^
*T*_2_	297.18^e^	299.23^g^	299.48^h^	1.25^g^	7.14^d^
*T*_3_	297.43^d^	299.48^f^	306.15^f^	0.77^h^	10.00^b^
*T*_4_	297.18^e^	302.56^d^	311.79^e^	2.56^b^	8.22^c^
*T*_5_	297.69^c^	312.82^b^	330.25^d^	2.13^d^	14.64^a^
*T*_6_	298.21^b^	311.79^c^	345.64^c^	2.30^c^	9.64^b^
*T*_7_	305.38^a^	357.95^a^	401.79^a^	2.97^a^	15.36^a^
*T*_8_	298.21^b^	299.48^f^	364.87^b^	2.05^e^	9.64^b^

Level of significance	∗∗	∗∗	∗∗	∗∗	∗∗

CV (%)	0.004	0.007	0.004	2.165	4.774

*Note:* ∗∗ = 1% level of significance. a, b, c, d, e, f, g, h, i indicate significant differences among treatments based on statistical analysis. Specifically, mean separation was performed using the Least Significant Difference (LSD) test at *p*< 0.05 (Statistix 10). Values sharing the same letter are not significantly different, whereas values with different letters differ significantly at the 5% probability level.

Abbreviation: CV = coefficient of variation.

**Table 6:** Severity scale of rice blast.

Rating no.	Type with foliage damage	Resistant level
0	No lesion or spots	Resistant
1	1%–5% of spots on the leaves	Moderately resistant
2	6%–20% of spots on the leaves	Moderately resistant
3	21%–40% of spots on the leaves	Moderately susceptible
4	41%–60% of spots on the leaves	Moderately susceptible
5	Above 61% of spots on leaves	Susceptible

## Data Availability

The data that support the findings of this study are available upon request from the corresponding author. The data are not publicly available due to privacy or ethical restrictions.
